# Value of the prognostic nutritional index and weight loss in predicting metastasis and long-term mortality in nasopharyngeal carcinoma

**DOI:** 10.1186/s12967-015-0729-0

**Published:** 2015-11-19

**Authors:** Xiao-Jing Du, Ling-Long Tang, Yan-Ping Mao, Rui Guo, Ying Sun, Ai-Hua Lin, Jun Ma

**Affiliations:** Department of Radiation Oncology, Sun Yat-sen University Cancer Center, State Key Laboratory of Oncology in South China, Collaborative Innovation Center for Cancer Medicine, No. 651 Dongfeng Road East, Guangzhou, 510060 People’s Republic of China; Department of Medical Statistics and Epidemiology, School of Public Health, Sun Yat-sen University, No. 74 Zhongshan Road, Guangzhou, 510060 China

**Keywords:** Prognostic nutritional index, Weight loss, Nasopharyngeal carcinoma, Predictive factor, Systemic inflammation response

## Abstract

**Background:**

To evaluate the influence of the progonistic nutritional index (PNI) and weight loss on metastasis and long-term mortality in nasopharyngeal carcinoma (NPC).

**Methods:**

We retrospectively reviewed 694 newly diagnosed patients with non-disseminated, biopsy-proven NPC. Survival analysis was used to evaluate the predictive value of PNI and weight loss.

**Results:**

Multivariate analysis demonstrated that a low pre-therapy PNI (< 55.0) was an independent predictor of poor overall survival (OS) (*P* = 0.012), distant metastasis-free survival (DMFS) (*P* = 0.011) and progression-free survival (*P* = 0.012). High weight loss (HWL, weight loss during treatment ≥10 %) was an independent predictor of poor OS (*P* = 0.001) and DMFS (*P* = 0.014). Advanced stage disease, female gender, chemotherapy, high white blood cell count, high serum globulin concentration and pre-therapy body-mass index were predictors of HWL.

**Conclusion:**

Pre-therapy PNI and weight loss have significant predictive value for metastasis and mortality in patients with NPC.

## Background

Nasopharyngeal carcinoma (NPC) is one of the most commonly diagnosed malignancies in Southeast Asia, with an annual incidence of 30–80 per 10,000 populations [1]. Radiotherapy (RT), alone or in combination with chemotherapy, is the mainstay treatment for NPC [2]. Advances in diagnostic imaging, radiotherapeutic techniques and chemotherapy regimens have provided significant survival benefits for locally advanced NPC [3]. However, 20 % of the patients with advanced disease still develop metastases even after they are found to be disease free [2]. Metastatic NPC is associated with poor prognosis, with a median survival period of approximately 17 months [4]. Identifying the factors that predict the aggressiveness of NPC and the associated mortality is of utmost importance.

Variations in the outcomes of cancer patients are not solely determined by the characteristics of the tumour but also by the host response factors [5, 6]. The systemic inflammatory response is a non-specific response secondary to tumour necrosis or local tissue damage caused by tumour-host cell interactions [7]. As part of the systemic inflammatory response, pro-inflammatory cytokines and growth factors are released, some of which cause metabolic disturbances and the loss of lean tissue and promote tumour growth [8]. Recently, there has been increasing evidence to suggest that systemic inflammatory response is of prognostic value in patients with various types of cancers: it can predict survival, independent of the disease stage, in advanced cancers [5, 7]. The host systemic inflammatory response can be assessed by examining changes in the circulating concentrations of acute-phase proteins and other cellular components, such as neutrophils, lymphocytes, monocytes and platelets [9].

The prognostic nutritional index (PNI), which reflects the albumin concentration and lymphocyte count, is a novel systemic inflammation-based prognostic score [9]. It was first introduced by Onodera et al. to assess the immunological and nutritional status of patients undergoing gastrointestinal surgery [10]. Research conducted over the last decade or so has demonstrated associations between a low PNI and poor survival in several types of cancers [11–18]. The impact of PNI on metastasis and mortality in NPC has not yet been addressed.

Weight loss is commonly observed in cancer patients and is associated with increased morbidity and mortality. The prevalence of weight loss is estimated to be between 40 and 80 %, with a particularly high incidence in patients with head and neck cancer [6]. Significant weight loss occurs frequently during radiotherapy in NPC patients [20–22]; it increases the risk of treatment gaps [23] and is associated with poor survival [24]. Accumulative data have demonstrated that weight loss is not just a nutritional indicator that reflects reduced intake or nutritional imbalance. The presence of systemic inflammatory response has recently been shown to be associated with weight loss in cancer patients [5]. Unlike other prognostic factors, weight loss can be treated as a therapeutic target that can be managed through available nutritional support. Based on the fact that early nutritional management is associated with better prognosis in head and neck cancer [25], the identification of risk factors for weight loss, particularly pre-therapy risk factors, is of great importance for early intervention. In this study, we re-examined weight loss in NPC and its effect on the clinical outcome, given the recent advances in the treatment of NPC.

The aims of the present study were (1) to evaluate the predictive value of PNI, weight loss and other selective markers of systemic inflammatory response for survival, and (2) to determine whether certain pre-therapy clinical factors could predict weight loss during treatment in NPC patients.

## Methods

### Patients

We conducted a retrospective study using the in-patient medical records of all newly diagnosed patients with histologically proven, non-disseminated NPC treated at Sun Yat-sen University Cancer Center (Guangzhou, China) between January 2003 and December 2006. In all, 719 cases were evaluated, among which 25 (3.5 %) were subsequently eliminated, including 18 (2.5 %) patients with incomplete laboratory data, 5 (0.7 %) who were unable to complete the prescribed treatment, and 2 (0.3 %) with other malignancies in addition to NPC. Thus, 694 patients were finally included in the analysis. The study was approved by the Institutional Review Board of Sun Yat-sen University Cancer Center. As this was a retrospective analysis of routine data, we requested and were granted a waiver of individual informed consent from the ethics committee. Patient records/information was anonymized and de-identified prior to analysis.

### Treatment

All the patients underwent a pretreatment baseline evaluation including complete medical history, physical and neurological examinations, hematology and biochemistry profiles, MRI scan of the neck and nasopharynx, chest radiography, and abdominal sonography. The treatment plans were determined according to standard protocols depending on the tumor stage and general health of the patient. All the patients were treated with continuous definitive radiotherapy with daily fractions of 2.0 Gy five times per week using a linear accelerator (6–8 MV). The radiation dose ranges in the nasopharynx, lymph node-positive area and lymph node-negative area were 60–80, 60–70 and 50–60 Gy, respectively. In total, 302/694 (43.5 %) patients received precise radiotherapy, of whom 279 (40.2 %) received intensity-modulated radiotherapy and 23 (3.3 %) received 3-dimensional conformal radiotherapy. Induction or adjuvant chemotherapy consisted of three cycles of cisplatin with 5-fluorouracil, or cisplatin with taxanes every 3 weeks. Concurrent chemotherapy consisted of cisplatin every 3 weeks or cisplatin weekly. In total, 479/694 (69.0 %) patients underwent chemotherapy. Most of them (376/479, 78.5 %) had advanced stage disease (classified as T3–T4 and/or N2–N3). When possible, salvage treatments (including afterloading, surgery, and chemotherapy) were provided for residual disease or disease relapse. Participants were on 100 % oral intake at the time of the study, and no patient used any form of enteral tube feeding or total parenteral nutrition. The diet of the patients was not monitored in any way and was based on their choice, motivation, and ability.

### Data collection

Data were gathered from medical records, including age; sex; body weight and height; pre-therapy laboratory counts of white cells, neutrophils, lymphocytes, hemoglobin and platelets; bilirubin, alkaline phosphatase, albumin and globulin concentration; pathological types; clinical stage; and type of treatment. The neutrophil-to-lymphocyte ratio (NLR) was calculated by dividing the neutrophil count by the lymphocyte count. The platelet-to-lymphocyte ratio (PLR) was calculated by dividing the platelet count by the lymphocyte count. PNI was calculated using the following formula: serum albumin (g/L) + 0.005 × total lymphocyte count/μL [9]. Weight loss during treatment was calculated as follows: (weight at the baseline visit) − (weight at the end of treatment). The end of treatment was defined as completion of the entire prescribed treatment plan, for example, the end of adjuvant chemotherapy for concurrent chemo-RT plus adjuvant chemotherapy. Three groups were defined according to the percentage of weight loss: <5 % = low weight loss (LWL); 5–10 % = moderate weight loss (MWL); and ≥10 % = high weight loss (HWL). The patients were also classified into four groups according to their pre-therapy body mass index (BMI, kg/m^2^) according to the WHO recommendations for Asian populations [26]: <18.5 kg/m^2^ = underweight; 18.5–22.99 kg/m^2^ = normal weight; 23.0–27.49 kg/m^2^ = overweight, and ≥ 27.5 kg/m^2^ = obese.

### Follow-up

After completion of the therapy, the patients were examined every 3 months during the first 2 years, and every 5 months thereafter for up to 6 years or until death. The end of the follow-up was February 28, 2013. The median follow-up period was 88 months (range 5–123 months). None of the patients were lost to follow-up. The following end points (time to the first defining event) were assessed: distant metastasis-free survival (DMFS), overall survival (OS), progression-free survival (PFS) and local relapse-free survival (LRFS).

### Statistical analysis

All the calculations were performed using the Statistical Package for the Social Sciences, version 20.0 (SPSS, Chicago, IL, USA). Circulating marker levels were analysed as binary variables by using the median values of each marker as the cut-off levels, which is more than or equal to the median in the group with high values and less than the median in the group with low values. Pearson Chi-square (*χ*^2^) tests or Fisher’s exact test (when the expected number per cell was <5) was used to analyze categorical variables. Survival curves were drawn using the Kaplan–Meier method and compared using the log-rank test. Multivariate analysis using a Cox proportional hazards model was used to test independent significance by backward elimination of insignificant explanatory variables. For each categorical predictor, the reference category was defined as the category with the best prognosis: for example, LWL was the weight loss reference category. Two-tailed *P* values <0.05 were considered to indicate significance.

## Results

### Clinicopathological features and treatment outcomes

The clinicopathological characteristics of the 694 patients, including 517 (74.5 %) males and 177 (25.5 %) females, are presented in Table 1. The median age at diagnosis was 44 years (range 13–78 years). Based on the WHO criteria, 99.3 % of the patients had type II or III disease and 0.7 % had type I disease. All the patients were staged according to the 7th edition of the International Union against Cancer/American Joint Committee on Cancer (UICC/AJCC) staging system for NPC [27].

**Table 1:** Characteristics of the 694 patients with NPC

Variable	No. (%)	LWL/MWL (weight loss <10 %)	HWL (weight loss ≥10 %)	*P* value
Age, years				0.414
<50	473 (68.2)	350 (74.0)	123 (26.0)	
≥50	221 (31.8)	157 (71.0)	64 (29.0)	
Sex				0.001
Male	517 (74.5)	394 (76.2)	123 (23.8)	
Female	177 (25.5)	113 (63.8)	64 (36.2)	
T classification				0.001
T1–2	313 (45.1)	248 (79.2)	65 (20.8)	
T3–4	381 (54.9)	259 (68.0)	122 (32.0)	
N classification				0.009
N0	196 (28.2)	157 (80.1)	39 (19.9)	
N1–3	498 (71.8)	350 (70.3)	148 (29.7)	
Clinical stage				<0.001
I + II	235 (33.9)	191 (81.3)	44 (18.7)	
III + IV	459 (66.1)	316 (68.8)	143 (31.2)	
Chemotherapy				<0.001
No	215 (31.0)	186 (86.5)	29 (13.5)	
Yes	479 (69.0)	321 (67.0)	158 (33.0)	
White blood cell count, k/μL				0.035
<6.9	339 (48.8)	260 (76.7)	79 (23.3)	
≥6.9	355 (51.2)	247 (69.6)	108 (30.4)	
NLR				0.171
<2.3	360 (51.9)	271 (75.3)	89 (24.7)	
≥2.3	334 (48.1)	236 (70.7)	98 (29.3)	
Blood platelet count, k/μL				0.221
<221	342 (49.3)	257 (75.1)	85 (24.9)	
≥221	352 (50.7)	250 (71.0)	102 (29.0)	
PLR				0.347
<120.7	347 (50.0)	259 (74.6)	88 (25.4)	
≥120.7	347 (50.0)	248 (71.5)	99 (28.5)	
Hemoglobin, g/L				0.283
<144	335 (48.3)	251 (74.9)	84 (25.1)	
≥144	359 (51.7)	256 (71.3)	103 (28.7)	
PNI				0.301
<55.0	345 (49.7)	246 (71.3)	99 (28.7)	
≥55.0	349 (50.3)	261 (74.8)	88 (25.2)	
Alkaline phosphatase, U/L				0.337
<68	340 (49.0)	254 (74.7)	86 (25.3)	
≥68	354 (51.0)	253 (71.5)	101 (28.5)	
Bilirubin, μmol/L				0.859
<4.1	345 (49.7)	251 (72.8)	94 (27.1)	
≥4.1	349 (50.3)	256 (73.4)	93 (26.6)	
Albumin, g/L				0.399
<45.4	338 (48.7)	242 (71.6)	96 (28.4)	
≥45.4	356 (51.3)	265 (74.4)	91 (25.6)	
Globulin, g/L				0.009
<30.2	339 (48.8)	263 (77.6)	76 (22.4)	
≥30.2	355 (51.2)	244 (68.7)	111 (31.3)	
Body mass index, kg/m^2^				0.040
Underweight (<18.5)	66 (9.5)	57 (86.4)	9 (13.6)	
Normal weight (18.5–22.99)	305 (43.9)	225 (73.5)	80 (26.2)	
Overweight (23.0–27.49)	273 (39.3)	192 (70.3)	81 (29.7)	
Obese (≥27.5)	50 (7.3)	33 (66.0)	17 (34.0)	

*NLR* neutrophil/lymphocyte ratio, *PLR* platelet/lymphocyte ratio, *PNI* prognostic nutritional index, *LWL* low weight loss, *MWL* moderate weight loss, *HWL* high weight loss

The median PNI in our patient population was 55.0 (range 39.6–80.3). The median weight loss during treatment was 4.5 kg (range −2.0 to 16.0 kg), and the median percentage weight loss was 6.7 % (range −4.0 to 22.5 %). Additionally, 26.9 % of the patients experienced ≥10 % weight loss.

At the last follow-up, 185/694 patients (26.7 %) had tumor progression after the treatment, including 70 (10.1 %) patients who developed local or regional recurrence and 130 (18.7 %) who developed distant metastasis. By the end of the follow-up, 164/694 patients (23.6 %) died, including 150 (21.6 %) who died of NPC and 14 (2.0 %) who died of other causes. The 5-year OS, PFS, DMFS and LRFS rates were 82.6, 76.2, 83.6 and 89.9 %, respectively.

### Prognostic value of PNI and weight loss

**Fig. 1:**
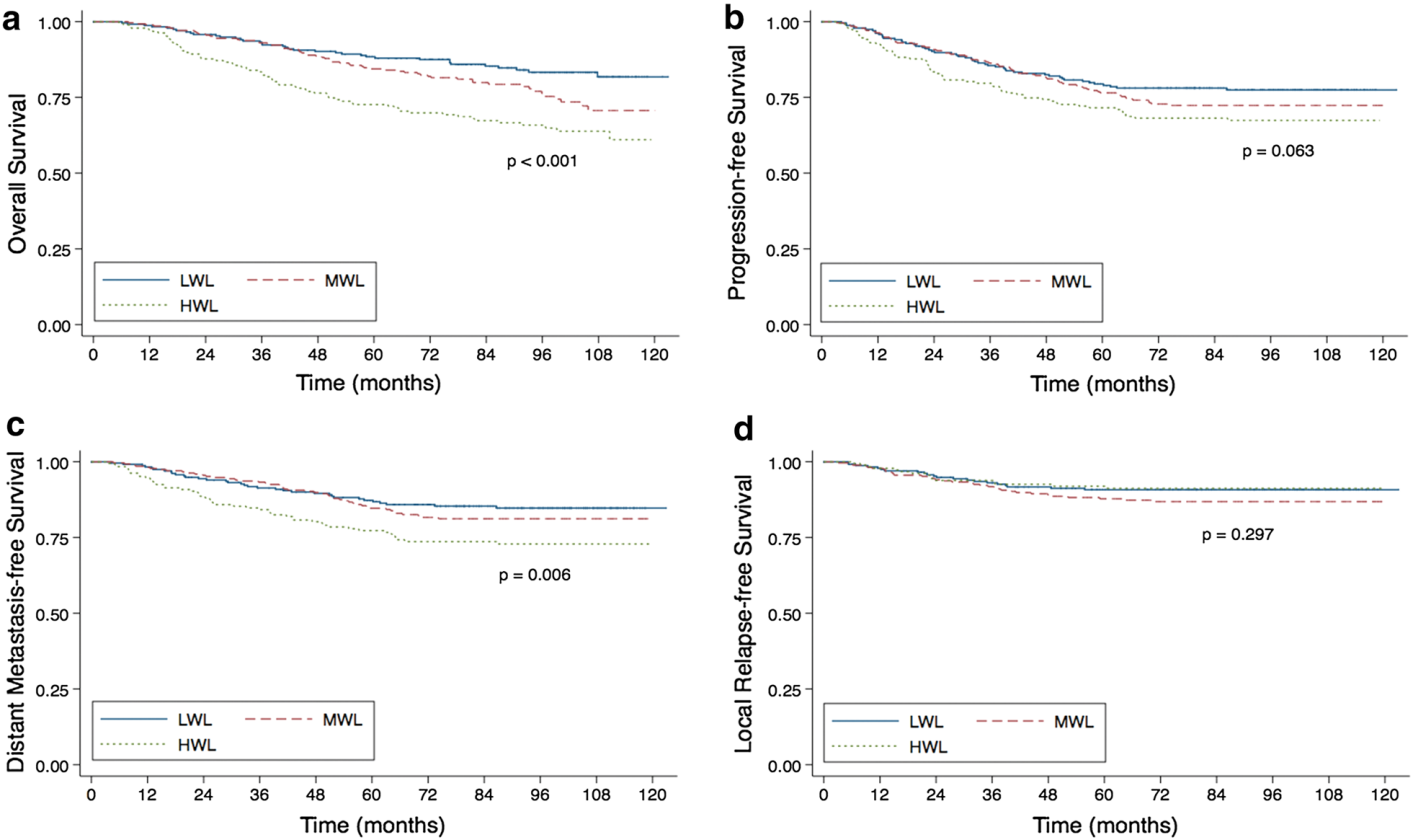
Weight loss related outcomes. Kaplan–Meier overall survival curves (**a**), progression-free survival curves (**b**), distant metastasis-free survival curves (**c**) and local relapse-free survival curves (**d**) for patients with NPC stratified by weight loss during treatment into low weight loss (LWL), moderate weight loss (MWL) and high weight loss (HWL) groups

**Fig. 2:**
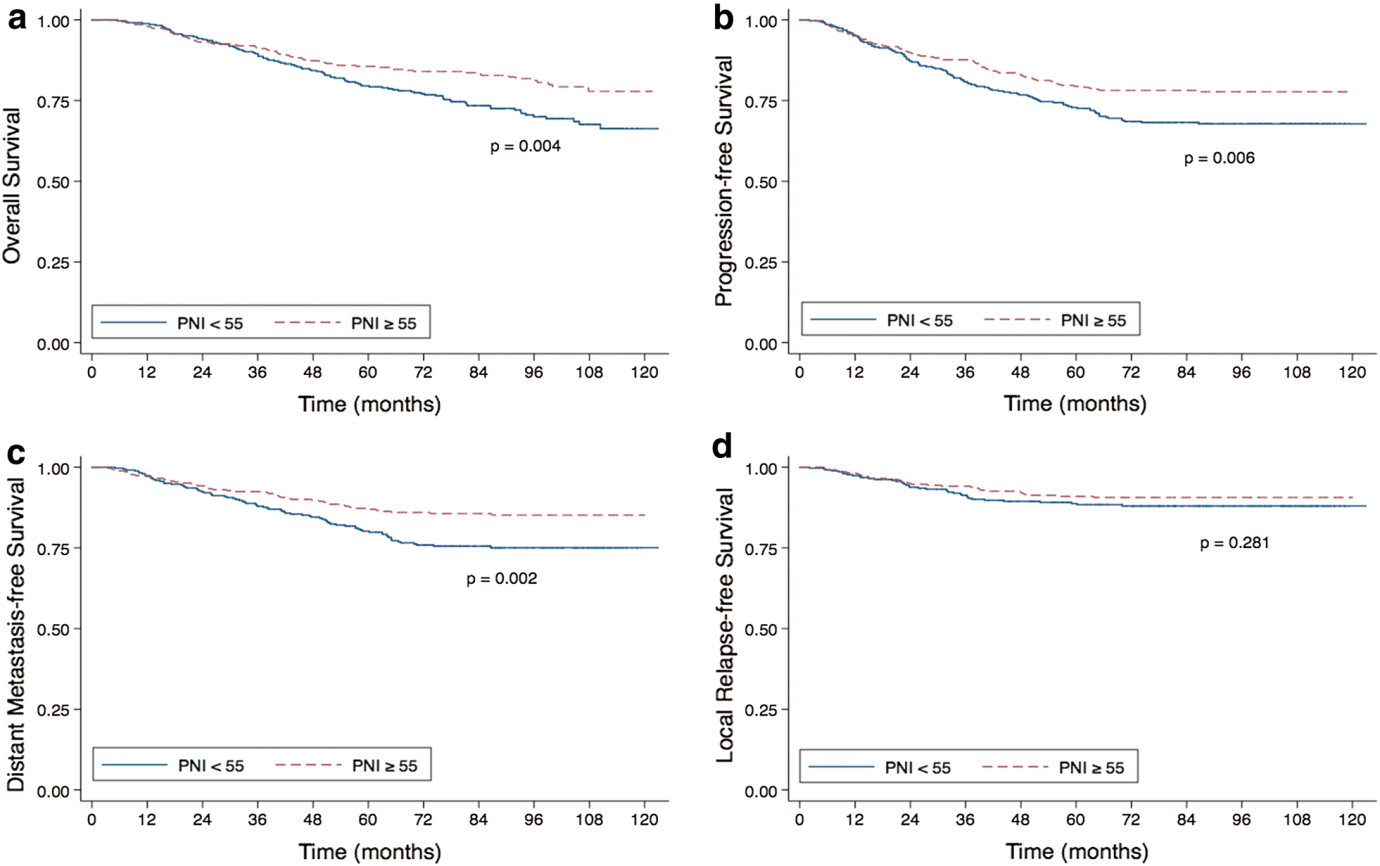
Prognostic nutritional index related outcomes. Kaplan–Meier overall survival curves (**a**), progression-free survival curves (**b**), distant metastasis-free survival curves (**c**) and local relapse-free survival curves (**d**) for patients with NPC stratified by prognostic nutritional index (PNI)

Univariate analysis identified pre-therapy PNI as a significant predictive factor for DMFS (*P* = 0.002), OS (*P* = 0.004) and PFS (*P* = 0.006). Weight loss was significantly associated with poor DMFS (*P* = 0.006) as well as OS (*P* < 0.001), and had a marginally significant influence on PFS (*P* = 0.063). However, both pre-therapy PNI and weight loss had no predictive value for LRFS (*P* = 0.281; *P* = 0.297). The Kaplan–Meier curves are shown in Figs. 1 and 2.

Multivariate analysis for DMFS, OS and PFS was performed to adjust for various prognostic factors. The following parameters were included in the Cox proportional hazards model: age (≥50 vs. <50 years), gender (male vs. female), T classification (T3–4 vs. T1–2), N classification (N1–3 vs. N0), chemotherapy (yes vs. no), pre-therapy BMI, pre-therapy white blood cell count (≥6.9 vs. <6.9 k/μL), pre-therapy NLR (≥2.3 vs. <2.3), pre-therapy PLR (≥120.7 vs. <120.7), pre-therapy blood platelet count (≥221 vs. <221 k/μL), pre-therapy hemoglobin (<144 vs. ≥144 g/L), pre-therapy PNI (<55.0 vs. ≥55.0), pre-therapy alkaline phosphatase (≥68 vs. <68 U/L), pre-therapy bilirubin (≥4.1 vs. <4.1 μmol/L), pre-therapy serum globulin concentration (≥30.2 vs.<30.2 g/L), and the percentage weight loss groups. The serum albumin concentration was excluded, as it was used in the calculation of PNI.

Consistent with the results of the univariate analysis, pre-therapy PNI was found to be an independent predictive factor for DMFS, OS and PFS (*P* = 0.011, 0.012 and 0.012, respectively). As weight loss increased, the risk of metastasis and death increased (*P* = 0.038 and 0.002 for DMFS and OS, respectively). Compared to LWL, HWL had a strong adverse effect on DMFS and OS (*P* = 0.014; *P* = 0.001). Being underweight at the baseline was significantly associated with DMFS (*P* = 0.008), compared to having normal weight. Besides, the serum alkaline phosphatase concentration was significantly associated with DMFS, OS and PFS. The serum globulin concentration was a significant prognostic factor for DMFS and OS. T classification was identified as an independent predictive factor for PFS. Furthermore, N classification was identified as an independent predictive factor for PFS and DMFS. In contrast, age, gender, chemotherapy, white blood cell count, NLR, PLR, hemoglobin, blood platelet count and bilirubin were not independent predictive factors for any survival endpoint according to the results of the multivariate analysis (Table 2).

**Table 2:** Multivariate analysis of prognostic factors in the patients with nasopharyngeal carcinoma (N = 694)

Variable	DMFS			OS		PFS
	*P* value	HR (95 % CI)	*P* value	HR (95 % CI)	*P* value	HR (95 % CI)
Age (≥50 vs. <50 years)						
Sex (male vs. female)						
T classification (T3–4 vs. T1–2)			0.051	1.392 (0.998–1.942)	0.028	1.410 (1.038–1.915)
N classification (N1–3 vs. N0)	0.005	1.903 (1.215–2.982)	0.054	1.439 (0.994–2.085)	0.005	1.663 (1.165–2.374)
Chemotherapy (yes vs. no)						
White blood cell count (≥6.9 vs. <6.9 k/μL)						
NLR (≥2.3 vs. <2.3)						
Blood platelet count (≥221 vs. <221 k/μL)	0.078	1.373 (0.965–1.954)				
PLR (≥120.7 vs. <120.7)						
Hemoglobin (<144 vs. ≥144 g/L)						
PNI (<55.0 vs. ≥55.0)	0.011	1.603 (1.116–2.303)	0.012	1.501 (1.095–2.058)	0.012	1.460 (1.088–1.961)
Alkaline phosphatase (≥68 vs. <68 U/L)	0.002	1.773 (1.233–2.549)	0.003	1.626 (1.177–2.247)	<0.001	1.711 (1.266–2.313)
Bilirubin (≥4.1 vs. <4.1 μmol/L)						
Globulin (≥30.2 vs. <30.2 g/L)	0.032	1.485 (1.034–2.134)	0.041	1.396 (1.013–1.924)		
Body mass index	0.017					
Underweight (<18.5 kg/m^2^)	0.008	2.060 (1.212–3.501)				
Normal weight (18.5–22.99 kg/m^2^)		1				
Overweight (23.0–27.49 kg/m^2^)	0.297	1.230 (0.834–1.812)				
Obese (≥27.5 kg/m^2^)	0.185	0.534 (0.211–1.351)				
Weight loss, %	0.038		0.002			
LWL (less than 5.0 %)		1		1		
MWL (5.0–9.99 %)	0.343	1.243 (0.792–1.950)	0.082	1.437 (0.955–2.161)		
HWL (10 % or more)	0.014	1.787 (1.127–2.834)	0.001	2.053 (1.362–3.095)		

*NLR* neutrophil/lymphocyte ratio, *PLR* platelet/lymphocyte ratio, *PNI* prognostic nutritional index, *LWL* low weight loss, *MWL* moderate weight loss, *HWL* high weight loss, *OS* overall survival, *OS* overall survival, *PFS* progression-free survival, *DMFS* distant metastasis-free survival, *95* *% CI* 95 % confidence interval, *HR* hazard ratio

### Association of weight loss with pre-therapy clinical features

The distribution of percentage weight loss differed significantly when the patients were stratified by T classification, N classification and clinical stage. The HWL group contained a significantly higher number of patients with T3-4 and N1-3 stage disease than those with T1-2 (*P* = 0.001) and N0 (*P* = 0.009) stage disease. Moreover, 44/235 (18.7 %) and 143/459 (31.2 %) of the patients with stage I + II and stage III + IV disease respectively experienced more than 10 % weight loss during treatment (*P* < 0.001). Female patients and patients who underwent chemotherapy had higher weight loss during treatment than male patients (*P* = 0.001) and patients who did not undergo chemotherapy (*P* < 0.001). Additionally, the HWL group contained a significantly higher number of patients with a high pre-therapy white blood cell count and high pre-therapy serum globulin concentration than patients with a low white blood cell count (*P* = 0.035) and low serum globulin concentration (*P* = 0.009). Moreover, the proportion of patients in the HWL group significantly increased along with the increase in pre-therapy BMI (Table 1).

## Discussion

The findings in this study indicate that PNI and weight loss are associated with tumor progression, metastasis and long-term mortality in patients with NPC. Moreover, higher pre-therapy BMI, white blood cell count, serum globulin level, female gender, chemotherapy, and advanced stage disease were predictive of greater weight loss during treatment.

PNI is calculated from albumin concentration and lymphocyte count. Serum albumin is known to correlate with systemic inflammation through high levels of pro-inflammatory cytokines. Albumin may help to stabilize cell growth and DNA replication, buffer a variety of biochemical changes, and maintain sex hormone homeostasis to protect against cancers [28]. Growing numbers of studies have demonstrated associations between a low serum albumin level and an increased severity of disease, a high risk of disease progression and poor survival in cancer patients [28]. Lymphocytes are crucial components of adaptive immune system, which is always suppressed in tumours through several pathways, including inhibition of dendritic cell differentiation and activation, infiltration of regulatory T cells and tumour-associated macrophages [7, 8, 29]. Infiltrating lymphocytes have been reported to represent an effective antitumour cellular immune response. A low peripheral lymphocyte level may indicate a poor lymphocyte-mediated immune response to tumour and suggest a poor prognosis [29]. Previous studies have shown that a high lymphocyte count was associated with improved clinical outcome in rectal cancer [30] and pancreatic ductal adenocarcinoma [31]. High peripheral lymphocyte percentage was also demonstrated to be associated with a long survival of patients with NPC [29].

Consistent with our results for NPC, the prognostic value of PNI has been validated in other tumors in several published studies, including pancreatic cancer [11], gastric carcinoma, esophageal carcinoma [13], malignant pleural mesothelioma [14], hepatocellular carcinoma [15, 16], colorectal cancer [17], and renal cell cancer [18]. To the best of our knowledge, this is the first study to specifically focus on the predictive value of PNI in NPC. In the present study, it would appear that PNI is superior to other systemic inflammation-based factors, in particular, the white blood cell count, NLR and PLR, in predicting survival in NPC.

Another prognostic factor for metastasis and mortality in NPC that we identified in this study is serum globulin. High levels of globulins are attributable to increased accumulation of acute-phase proteins and immunoglobulins, as well as other serum proteins, and all these changes are reflective of an inflammatory state. Besides, elevated levels of serum alkaline phosphatase have been reported to predict disease progression and poor outcome in some malignancies, including NPC [19]. It was considered as an indicator of distant micrometastases in other organs such as bone [19].

Another factor that this study examined was weight loss, which has been shown to affect the clinical outcome in NPC patients. Ng et al. reported that the mean percentage weight loss during radiotherapy in 38 patients with NPC from Hong Kong was 10.8 %, and that 55 % of the patients experienced ≥10 % weight loss by the end of the treatment [20]. Qiu et al. conducted a prospective study of 159 patients with NPC and observed a median weight loss of 6.9 kg (range 2.1–12.6 kg) [21]. Oates et al. reported that the median weight loss during concurrent chemoradiotherapy in 14 patients with NPC was 8.2 kg (range 2.3–13.9 kg), which represented a percentage weight loss range of 4–17 % [22]. Further, Shen et al. conducted a retrospective study in 2433 NPC patients and reported that high weight loss during radiotherapy (weight loss ≥5 %) was indepentently associated with poor survival [24]. In the present study, we evaluated weight loss during the entire treatment procedure and found that high weight loss (weight loss ≥10 %) was still a predictor of metastasis and survival.

We speculate that there are three possible reasons why weight loss significantly associates with mortality. First, percentage weight loss is a commonly used tool in assessment of recently developed malnutrition. Malnutrition can weaken a number of human defense mechanisms, including anatomic barriers, cellular and humoral immunity, and phagocyte function [5–7], thus promoting susceptibility to infection and further compromising the response to malignancy. Second, weight loss may cause treatment interruption and decrease treatment tolerance, thus affecting therapeutic efficacy [23]. Additionally, weight loss is also an indicator of systemic inflammatory response, that encompasses a variety of physiologic alterations facilitating tumour development, invasion, and metastasis [5, 8].

Weight loss during NPC treatment is partially due to oral mucositis induced by radiotherapy and chemotherapy. Oral mucositis is considered to be the most important acute side effect in almost all patients undergoing radiotherapy of the head and neck [32]. The oral lesions may cause dysphagia with solid and liquid food, dysarthria and odynophagia, and thus directly reduce food intake. Effective management of oral mucositis may minimize the symptoms, improve the nutritional status, allow for more effective cancer treatment and improve patient survival [32].

Many factors affect weight loss in cancer patients. Consistent with the findings of Qiu et al. [21], we observed that high weight loss was associated with heavier tumor burden and chemotherapy. Patients treated with chemotherapy are more likely to have advanced stage disease. Despite the encouraging results achieved by multimodal therapy in terms of tumor control, aggressive chemoradiotherapy can lead to more serious acute toxicities, which directly impact the ability to eat [32]. We also found that female patients were more likely to experience high weight loss than males. Similar findings have been reported in head and neck cancer [33]. Further, a recent study showed that female patients with NPC tended to experience anxiety before treatment, lacked a positive coping mode, and experienced poor quality of life after radiotherapy [34]. We speculated that poorer treatment compliance and tolerance among female patients may account for their greater weight loss. In the present study, higher pre-therapy BMI was associated with higher weight loss in NPC, which has also been reported by Qiu et al. [21]. A patient with a higher pre-therapy BMI might have more weight to lose and show strong resistance to malnutrition. Furthermore, a high white blood cell count and high serum globulin concentration were shown to be predictors of high weight loss. In keeping with our results, there is good consistent evidence that the presence of systemic inflammatory response is associated with increased weight loss, elevated resting energy expenditure, loss of lean tissue and functional decline [5]. These findings may suggest the effectiveness of anti-inflammatory therapy in weight loss in cancer patients.

There are some limitations to the current study. First, the data were retrospectively analyzed. Second, PNI was only assessed at a single time point before the treatment. Thus, the relationship between the kinetics of PNI and its prognostic effect in NPC would be of considerable interest. Third, there were no standard criteria for nutritional support in patients undergoing oncotherapy during the time period of the study. Therefore, insufficient information on food intake and nutritional status during the treatment is available for further analysis of weight loss.

## Conclusion

The pre-therapy PNI and weight loss represent important prognostic factors for evaluating the long-term metastasis and mortality in patients with NPC. This study offers good insight into the relationship between the systemic inflammatory response and survival in patients with NPC. The results would also be highly useful with regard to selecting patients for preventive measures before treatment.
